# Determination of the effect of pre-mating weight and placental characteristics on birth weight in Karayaka sheep

**DOI:** 10.5194/aab-66-335-2023

**Published:** 2023-11-17

**Authors:** Samet Urun, Emre Şirin

**Affiliations:** Department of Agricultural Biotechnology, Faculty of Agriculture, Kırşehir Ahi Evran University, 40100 Kırşehir, Türkiye

## Abstract

The aim of this study was to determine the effect of the pre-mating weight and placental characteristics on birth weight. Data were collected from 62 Karayaka ewes and 70 Karayaka lambs. The placental characteristics considered were placental weight, placental area and the number of cotyledons. The Pearson correlation coefficient was used for statistical comparison and the determination of relationships between variables. In addition, correlation coefficients between live weights and placental characteristics were determined. The average birth weight (BW), pre-mating weight (PMW), placental weight (PW), placental area (PA) and cotyledon number (CN) values were 4.37 ± 0.70 kg, 50.22 ± 5.63 kg, 362.51 ± 118.42 g, 994.18 ± 312.76 cm ${}^{2}$ and 56.93 ± 8.06, respectively. BW had positive correlations with PMW (0.147), birth type (BT) (0.643), PW (0.604), PA (0.323) and CN (0.161) ( P < 0.05). BW had negative correlations with maternal age (MA) ( -0.119 ) ( P < 0.05). PMW had positive correlations with maternal age (MA) (0.237) ( P < 0.05). PMW had negative correlations with S (sex) ( -0.003 ), PW ( -0.049 ), PA ( -0.067 ) and CN ( -0.080 ) ( P > 0.05).

## 1 Introduction

Lamb production is one of the most important yield values in sheep husbandry. For this reason, reproductive efficiency is as important as meat production and quality. In developing countries, different domestic sheep breeds that are adapted to the specific region are used in production. There are approximately 42 million sheep in Türkiye. An important component of these sheep are the fat-tailed native sheep breeds. The remaining animals are comprised of foreign sheep breeds and crossbreeds (Sen et al., 2021). The most important domestic sheep breeds in Türkiye are the Akkaraman, Morkaraman, Karayaka and Awassi. These native breeds constitute 80 % of the total sheep stock and have a high adaptability to poor environmental conditions (Gungor and Unal, 2020; Sirin et al., 2017; Yakan and Ünal, 2010).

**Figure 1:**
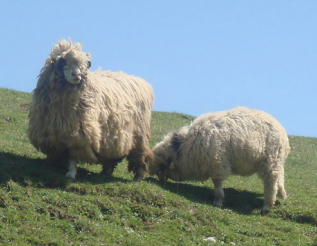
A photograph showing two Karayaka sheep.

The Karayaka sheep breed (Fig. 1) is one of the native breeds of Türkiye and is intensively produced in the Black Sea region. It is a carpet-wool breed that is also kept for meat production. The Karayaka breed is highly adaptable to adverse climatic conditions and poor pasture conditions. This native breed is widely produced in the provinces of Tokat, Amasya, Samsun, Ordu, Giresun and Sinop. The Karayaka breed has a high adaptability to the extremely rainy climate of the Black Sea region. The body colour of the Karayaka sheep breed is generally white with possible black and brown plaque on the head, ear, leg and body, although black and brown animals are occasionally seen (Ulutas et al., 2009).

The lamb birth weight is of great importance in sheep husbandry (Petrovic et al., 2011), as birth weight affects the development and mortality rates of lambs (Vatankhah and Taleb, 2009).

The placenta is a temporary tissue (only occurs during pregnancy) that is formed between the chorionic and uterine mucosa of the offspring for the development of the offspring. It is responsible for feeding the embryo and contributing to its development, regulating blood flow, removing metabolic waste products and gas, releasing hormones, and fulfilling the role of immunological barrier (Sen et al., 2013).

The structure of the placenta in sheep is multicotyledonary; this structure allows the placenta, where the foetus is located, to attach to the uterine endometrium (Sen et al., 2013). Therefore, placental growth, placental development and its nutrient transfer capacity play a central role in the determination of the prenatal growth trajectory of the foetus, resulting in alteration of birth-related traits, such as the birth weight, birth type and litter size (Sen and Onder, 2016). Placental characteristics are a valuable indicator of offspring mortality for small ruminants (Sen et al., 2013). Previous studies have reported that lamb survival is highly correlated with birth weight (De Barbieri et al., 2017); this is especially true for multiple births, which are associated with a high risk of pre-term birth and low birth weight (Schreurs et al., 2010).

Many existing studies have revealed a relationship between the birth weight and placental characteristics in sheep and goats. These publications have demonstrated a positive relationship of birth weight and placental characteristics (Echternkamp, 1993) with cotyledon number (CN) for calves (Konyalı et al., 2007) and of birth weight and placental characteristics with placental efficiency (Alkass et al., 2013; Ozyurek, 2019) for kids. In addition, placental weight is affected by the birth type (Kaulfuss et al., 2000). Placental characteristics and birth weight are also affected by the parity in sheep (Dwyer et al., 2005). A positive relationship between cotyledon weight and cotyledon efficiency in sheep has also been demonstrated (Ocak et al., 2014). In recent studies, determination of the total or average surface area of the cotyledons on the placenta instead of the number of cotyledons and cotyledon weight has been used as a new method (Özyürek ve Türkyılmaz, 2020). On the other hand, it has been determined that cotyledon sizes have an effect on the birth weight in lambs and kids (Ocak et al., 2015; Sen and Onder, 2016).

Placental characteristics are one of the important factors for maintaining pregnancy under adverse environmental conditions that may be experienced by pregnant animals. In addition, placental characteristics can be used as a selection criteria for reproductive performance. It is also known that there is a strong relationship between low birth weight and lamb death. Although studies have determined a relationship between birth weight and placental characteristics, no existing study, to our knowledge, has determined a relationship between pre-mating weight and birth weight. This is also the first study in which placental surface area has been used as a factor to determine the relationship between placental characteristics and birth weight. In this work, we aimed to determine the effect of pre-mating weight and placental characteristics on the birth weight of Karayaka sheep.

## 2 Materials and methods

The experimental animals used in this work were 2- to 5-year-old Karayaka sheep that were maintained at the Agricultural Research and Application Farm of Gaziosmanpaşa University, Tokat, Türkiye (40 ${}^{\circ }$ 31 ${}^{\prime }$ N, 36 ${}^{\circ }$ 53 ${}^{\prime }$ E; 650 m above sea level). The data were collected from 62 Karayaka sheep and 70 Karayaka lambs. Sheep were weighed before breeding, and their live weights were determined. In addition, the ages of the sheep were determined. Experimental groups were formed as follows: a single-birth group (54 sheep and 54 lambs), a twin-birth group (8 sheep and 16 lambs), and a single- and twin-birth (hereafter single + twin) group (62 sheep and 70 lambs). The birth weight, birth type (single or twin) and the sex of lambs were recorded within 12 h of lambing. Each ewe was left to deliver the placenta naturally, and placentas were collected from single and twin births immediately after delivery (approximately 2 h after the birth of the lamb); care was taken to ensure that any placental weights taken were of the total placenta, with any fluid being removed before weighing. The numbers of cotyledons and the weights of placenta were also determined and recorded. The areas of the placentas were determined by making measurements on a flat surface.

The formula used to determine the area of the placenta was as follows:

$$\text{placenta area}\;({\mathrm{cm}}^{2})\;=\;\pi \times \text{placenta width}\;\times \;\text{placenta length}.$$

The analysis of data was carried out in SPSS 17.0. Relationships between the placental characteristics and the pre-mating weight were determined with a Pearson correlation analysis at a 95 % confidence interval. The Fisher z -transformation test was applied to assess the significance of the differences between correlation coefficients.

The interpretation of the Pearson correlation coefficient was as follows:

-
r = 0.00–0.25 denoted a weak low relationship,
-
r = 0.26–0.49 denoted a weak relationship,
-
r = 0.50–0.69 denoted a moderate relationship,
-
r = 0.70–0.89 denoted a strong relationship,
-
r = 0.90–1.00 denoted a very strong relationship (Doymuş, 2016).

## 3 Results

The descriptive values determined in this study are given in Table 1. The birth weight was determined to be 4.61, 3.55 and 4.37 kg with respect to single-birth lambs, twin-birth lambs and the average, respectively. The pre-mating weight was determined to be 49.77, 51.75 and 50.22 kg with respect to single-birth lambs, twin-birth lambs and the average, respectively. The placenta weight, placenta area and cotyledon number were determined to be 397.98, 242.81 and 362.51; 1051.18, 801.78 and 994.18 cm ${}^{2}$ ; and 57.44, 55.19 and 56.93 with respect to single-birth lambs, twin-birth lambs and the average, respectively.

**Table 1:** Descriptive values for single births, twin births and their average.

Variables	n	Min	Max	$\bar{X}$	$\bar{Sx}$	SD
BW	54	3.20	6.00	4.61	0.075	0.55
	16	2.40	4.00	3.55	0.120	0.48
	70	2.40	6.00	4.37	0.083	0.70
PMW	54	39.80	66.55	49.77	0.809	5.94
	16	45.65	57.80	51.75	1.051	4.20
	62	39.80	66.55	50.22	0.673	5.63
PW	54	202.00	872.00	397.98	14.685	107.91
	16	156.00	410.00	242.81	14.886	59.54
	70	156.00	872.00	362.51	14.150	118.42
PA	54	399.00	1984.00	1051.18	39.429	289.74
	16	465.00	1460.50	801.78	79.908	319.62
	70	399.00	1984.00	994.18	37.380	312.76
CN	54	40.00	73.00	57.44	1.058	7.77
	16	41.00	67.00	55.19	2.253	9.01
	70	40.00	73.00	56.93	0.964	8.06

For each variable listed in the table, the first row presents values for single births, the second row presents values for twin births and the third row presents average values. The variables are as follows: BW – birth weight (kg); PMW – pre-mating weight (kg); PW – placenta weight (PW); PA – placenta area (cm ${}^{2}$ ); CN – cotyledon number.

The correlation coefficients of relationships between the lamb birth weight and other parameters are given in Table 2. The correlation coefficient ( r ) values between BW and PMW in single + twin, single-birth and twin-birth lambs were 0.147, 0.275 and 0.592 ( P < 0.05), respectively. The r values between BW and MA in single + twin, single-birth and twin-birth lambs were -0.119 , 0.013 and 0.203 ( P > 0.05), respectively. The r value between BW and birth type (BT) was 0.643 ( P < 0.05). The r values ( r ) between BW and PW, PA and CN in single + twin, single-birth and twin-birth lambs were 0.604, 0.402 and 0.324; 0.323, 0.166 and 0.83; and 0.161, 0.064 and 0.285 ( P < 0.05), respectively.

**Table 2:** Correlation coefficients between lamb birth weight and other parameters.

X	Y	r (single + twin)	r (single)	r (twin)
PMW	BW	$r_{i}$ 0.147	$r_{i}$ 0.275 ${}^{*}$	$r_{i}$ 0.592 ${}^{*}$
MA	BW	$r_{i}-0.119$	$r_{i}$ 0.013	$r_{i}$ 0.203
PW	BW	$r_{i}$ 0.604 ${}^{*}$	$r_{i}$ 0.402 ${}^{*}$	$r_{i}$ 0.324
PA	BW	$r_{i}$ 0.323 ${}^{*}$	$r_{i}$ 0.166	$r_{i}$ 0.083
CN	BW	$r_{i}$ 0.161	$r_{i}$ 0.064	$r_{i}$ 0.285

${}^{*}$ Values are significant at the P < 0.05 level. The variables used in the table are as follows: PMW – pre-mating weight; BW – birth weight; MA – maternal age; PW – placenta weight; PA – placenta area; CN – cotyledon number.

The correlation coefficient matrix of single-birth and twin-birth lambs is given in Table 3. The coefficient of the relationship between the BW of single-birth and twin-birth lambs with PW (0.604) and PA (0.323) was found to be very significant ( P < 0.01). The coefficient of the relationship between the PW of single-birth and twin-birth lambs with the PA (0.421) was found to be very significant ( P < 0.01). The coefficient of the relationship between the PA of single-birth and twin-birth lambs with the CN (0.243) was found to be significant ( P < 0.05).

The correlation coefficient matrix of single-birth lambs is given in Table 4. The coefficient of the relationship between the BW of single-birth lambs with the PW (0.402) was found to be very significant ( P < 0.01). The coefficient of the relationship between the BW of single-birth lambs with the PMW (0.275) was found to be significant ( P < 0.05). The coefficient of the relationship between the PW of single-birth lambs with the PA (0.351) was found to be very significant ( P < 0.01).

The correlation coefficient matrix of twin-birth lambs is given in Table 5. The coefficient of the relationship between the BW of twin-birth lambs with the PMW (0.592) was found to be significant ( P < 0.05). The coefficient of the relationship between the PMW of twin-birth lambs with the MA (0.562) was found to be significant ( P < 0.05).

**Table 3:** Correlation coefficient matrix of single-birth and twin-birth (single + twin) lambs.

	BW	PMW	MA	S	PW	PA	CN
BW	1	0.147	-0.119	0.113	0.604 ${}^{**}$	0.323 ${}^{**}$	0.161
PMW		1	0.237 ${}^{*}$	-0.003	-0.049	-0.067	-0.080
MA			1	-0.233	-0.122	-0.115	0.175
S				1	0.007	0.130	-0.044
PW					1	0.421 ${}^{**}$	0.099
PA						1	0.243 ${}^{*}$
CN							1

${}^{*}$ Values are significant at the P ≤ 0.01 level. ${}^{**}$ Values are significant at the P ≤ 0.05 level. The variables used in the table are as follows: BW – birth weight; PMW – pre-mating weight; MA – maternal age; S – sex; PW – placenta weight; PA – placenta area; CN – cotyledon weight.

**Table 4:** Correlation coefficient matrix of single-birth lambs.

	BW	PMW	MA	S	PW	PA	CN
BW	1	0.275 ${}^{*}$	0.113	0.087	0.402 ${}^{**}$	0.166	0.064
PMW		1	0.170	0.021	0.027	-0.206	-0.097
MA			1	-0.263	0.088	-0.075	0.255
S				1	-0.156	0.038	-0.110
PW					1	0.351 ${}^{**}$	0.074
PA						1	0.157
CN							1

${}^{*}$ Values are significant at the P ≤ 0.01 level. ${}^{**}$ Values are significant at the P ≤ 0.05 level. The variables used in the table are as follows: BW – birth weight; PMW – pre-mating weight; MA – maternal age; S – sex; PW – placenta weight; PA – placenta area; CN – cotyledon weight.

**Table 5:** Correlation coefficient matrix of twin-birth lambs.

	BW	PMW	MA	S	PW	PA	CN
BW	1	0.592 *	0.203	0.041	0.324	0.083	0.285
PMW		1	0.562 ${}^{*}$	-0.046	0.172	0.014	0.073
MA			1	0,048	-0.182	0.451	0.122
S				1	0.331	0.098	0.111
PW					1	0.058	-0.139
PA						1	0.383
CN							1

${}^{*}$ Values are significant at the P ≤ 0.01 level. ${}^{**}$ Values are significant at the P ≤ 0.05 level. The variables used in the table are as follows: BW – birth weight; PMW – pre-mating weight; MA – maternal age; S – sex; PW – placenta weight; PA – placenta area; CN – cotyledon weight.

## 4 Discussion

This study was conducted to determine the effects of the pre-mating weight and placental characteristics on the birth weight in Karayaka sheep. Placental traits are one of the main causes of the postnatal death of offspring in goats and sheep (Dwyer et al., 2005). A past study reported that the postnatal viability of newborns was highly correlated with placental growth and development during pregnancy (Mellor and Stafford, 2004). The relationship between the BW and PW was found to be significant. The relationship between the BW and PW was not found to be significant in prior studies conducted on sheep (Ocak et al., 2013; Özyürek and Türkyilmaz, 2020). According to these results, the increase in the PW in Karayaka sheep also increases the BW. The relationship between the BW and PA was found to be significant (single + twin). No other previous studies using PA as a characteristic were found. Therefore, this is the first study in which the PA has been used as a criterion in sheep. A significant relationship was found between the PA and CN. Therefore, as the area of the placenta increases, the number of cotyledons connecting the placenta to the uterine endometrium increases. In addition, the relationship between the PA and PW was found to be very important. Accordingly, if the placental area grows, the weight of the placenta also increases. Placental size has a crucial role in determining the prenatal growth trend of the offspring and, consequently, the birth weight and postnatal survival (Sen et al., 2013). Therefore, it is important to evaluate the placental area as a criterion.

No previous studies showing a relationship between pre-breeding weight and lamb birth weight and placental characteristics were found. Thus, this study has an important place in this respect. The relationship between the PMW and placental characteristics was not found to be significant. In mature ewes, positive relationships between the live weight and both the birth weight and growth rate of lambs have been reported (Gibb and Treacher, 1980). The relationship between the PMW and BW was significant in this study. Positive effects of hogget live weight at mating have been found on the following parameters: the proportion of hoggets displaying oestrus (McMillan and Moore, 1983); the ovulation rate, in some breeds only (Meyer and French, 1979); the conception rate (McMillan and Moore, 1983); and the lambing percentage (Kenyon et al., 2004). However, the effect of hogget live weight on the birth weight and growth rate of the resulting lamb(s) is unknown. In this study, it was revealed that the birth weight in single-birth and twin-birth Karayaka lambs increased as the pre-mating weight increased.

## 5 Conclusion

Birth weight is an important factor that affects both the survival of the lamb and its performance in the future. The pre-mating weight and placental characteristics have an effect on the birth weight. Many studies have determined that a relationship exists between the birth weight and placental characteristics in sheep. However, no existing study has determined the relationship between birth weight and placental area. In this respect, this is the first such study conducted on sheep. This work showed that the birth weight increases with increasing placental area. Therefore, placental area can be used as a criterion in animal breeding to increase the birth weight. In this work, it was determined that there was a limited relationship between the pre-mating weight and birth weight of single-birth and twin-birth lambs. In other words, as the pre-mating weight of the sheep increased, the birth weight of the lamb increased. However, it is necessary to determine the extent to which this relationship is valid with respect to the pre-mating weights of the sheep. In order to determine this situation clearly, it is thought that using the body condition score in sheep as a criterion for determining this relationship may provide more accurate results.

## Data Availability

Data supporting the findings of this study are available upon request from the corresponding author. The data are not publicly available due to privacy or ethical restrictions.
